# Multidimensional exploration of hydrogels as biological scaffolds for spinal cord regeneration: mechanisms and future perspectives

**DOI:** 10.3389/fbioe.2025.1576524

**Published:** 2025-04-23

**Authors:** Chenxi Han, Jiao Jiao, Chan Gong, Jiatao Li, Min Zhao, Xiao Lu

**Affiliations:** ^1^ Department of Rehabilitation, Jiangsu Province People’s Hospital, The First Affiliated Hospital of Nanjing Medical University, Nanjing, China; ^2^ School of Rehabilitation Medicine, Nanjing Medical University, Nanjing, China

**Keywords:** spinal cord injury, hydrogels, neural regeneration, spinal cord rehabilitation, immune microenvironment spinal cord injury, hydrogel, immune microenvironment

## Abstract

Spinal cord injury (SCI) is a severe condition that frequently leads to permanent disabilities and neurological dysfunction. Its progression is driven by a multifaceted pathophysiology, encompassing direct trauma, secondary injury cascades, and intricate cellular and molecular responses. While current therapies focus on alleviating symptoms and restoring functionality, achieving effective neural regeneration in the spinal cord continues to be a significant challenge. Hydrogels, recognized for their exceptional biocompatibility, conductivity, and injectability, have shown great potential as advanced scaffolds to support neuronal and axonal regeneration. Recently, these materials have attracted significant interest in the field of SCI rehabilitation research. This review concludes recent progress in hydrogel-based strategies for SCI rehabilitation, emphasizing their distinct properties, underlying mechanisms, and integration with bioactive molecules, stem cells, and complementary biomaterials. Hydrogels foster neuronal regeneration by providing a tailored microenvironment, while advanced features such as self-repair, electrical conductivity, and controlled drug release significantly enhance their therapeutic potential in experimental models. This review explores hydrogel technologies and their applications, underscoring their potential to address the challenges of SCI treatment and paving the way for future clinical implementation.

## 1 Introduction

Spinal cord injury (SCI) is a debilitating condition that often results in lifelong disability. Epidemiological studies estimate that over 15 million individuals globally are affected by SCI (Organization, 2024), with an annual incidence ranging from 20 to 50 cases per million people. (Rubiano et al., 2015; Lu et al., 202a). SCI is typically categorized into the primary and secondary phases of injury (Flack et al., 2022). Primary injury typically arises from direct trauma, such as traffic collisions or high falls, causing spinal fractures, dislocations, or compression. These events lead to vascular rupture, cell death, and direct damage to spinal neural tissue, resulting in varying levels of neurological dysfunction, with severe cases often presenting as paraplegia or quadriplegia (Alizadeh et al., 2019). Secondary injury arises as a downstream effect of primary trauma, characterized by localized hemorrhage, inflammation, and scar formation. These pathological processes significantly hinder neuronal regeneration and synapse formation, thereby delaying spinal cord repair and functional recovery (Beck et al., 2010). SCI outcomes are generally poor as prolonged immobility frequently leads to serious complications such as pressure ulcers, aspiration pneumonia, and deep vein thrombosis in the lower extremities (Organization, 2024). SCI imposes significant economic and psychological burdens on patients and their families (Xiao et al., 2019).

Until now, no definitive cure for SCI has been identified. Contemporary clinical treatments for SCI primarily consist of early surgical decompression, steroid therapy, perfusion strategies, pharmacotherapy, and rehabilitation interventions (Ahuja et al., 2017). Although surgical interventions can alleviate spinal compression, they provide limited benefits for neural tissue repair and are insufficient to restore neurological function or prevent scar formation and the loss of neural conduction (Matsumoto et al., 2024). In pharmacotherapy, the blood-brain barrier (BBB) and blood-spinal cord barrier (BSCB) impede the direct delivery of drugs to the injury site or reduce their concentrations during transport, significantly diminishing their therapeutic effectiveness (Nazerian et al., 2023). Hydrogels, as innovative biomaterials, exhibit exceptional biocompatibility, conductivity, moldability, and favorable mechanical properties. These qualities make hydrogels suitable for applications as cell carriers, drug delivery systems (Ramos Ferrer and Sakiyama-Elbert, 2024), or scaffolds to promote neuronal and axonal growth (Sun et al., 2023), support neural regeneration, preventing scar formation, and protect the BSCB, thereby attracting significant attention in SCI treatment (Yin et al., 2024). This review offers an in-depth analysis of the classification, properties, applications, and mechanisms of hydrogels in SCI therapy, highlighting current challenges and suggesting directions for future development.

## 2 Classification and representative examples of hydrogels

**FIGURE 1:**
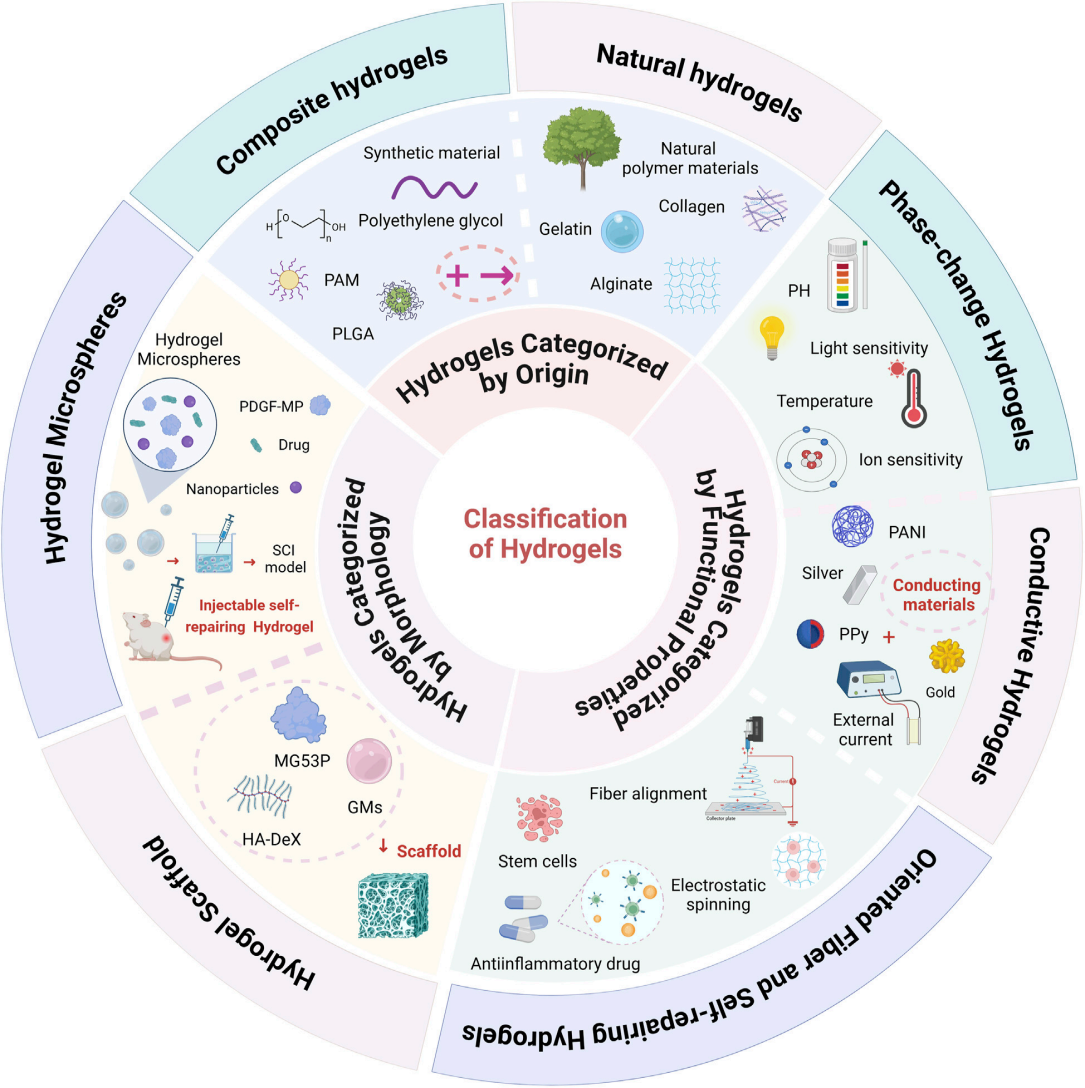
Classification of Hydrogels: Hydrogels are broadly classified into three main categories based on their source, morphology, and functional properties. In terms of their source, hydrogels are divided into natural hydrogels (e.g., gelatin, collagen) and composite hydrogels, which incorporate multiple components for enhanced performance. Based on morphology, hydrogels are categorized into hydrogel microspheres and hydrogel scaffolds. From a functional perspective, hydrogels include stimuli-responsive hydrogels (such as pH-responsive, thermosensitive, light-responsive, and ion-responsive types), conductive hydrogels, oriented fiber hydrogels, and self-healing hydrogels (Created in https://BioRender.com).

Hydrogels are typically composed of hydrophilic functional groups, such as hydroxyl (-OH) and amino (-NH_2_) groups, along with a crosslinked network. This structure enables them to absorb and retain a substantial amount of water, granting them high biocompatibility and biodegradability in physiological environments. Based on their synthesis method, hydrogels can be categorized into natural hydrogels and composite hydrogels (Prabakaran et al., 2024). In terms of morphology, they are classified into hydrogel microspheres and scaffold hydrogels. Additionally, hydrogels can be grouped according to their functional properties, including stimuli-responsive hydrogels (e.g., pH-responsive, thermosensitive, magnetically responsive, and light-responsive hydrogels), conductive hydrogels, as well as oriented fiber and self-healing hydrogels (Sun et al., 2023) (Figure 1).

### 2.1 Hydrogels categorized by origin

In SCI repair, hydrogels have gained considerable attention as promising biomaterials for tissue engineering applications. This is largely due to their high-water content, ability to replicate the ECM microenvironment, excellent biocompatibility, and customizable mechanical properties. Based on their origin, hydrogels are generally classified into natural and composite types (Wang et al., 202a). Although synthetic hydrogels play a critical role in materials science and tissue engineering, their standalone use in SCI repair remains relatively uncommon. More often, they are incorporated as part of composite hydrogels to enhance functionality and performance. Therefore, in this review, we group synthetic hydrogels within the category of composite hydrogels to better reflect current research directions in the field (Table 1).

**TABLE 1:** Hydrogels categorized by origin.

Classification	Composition	Crosslinking method	Biological model	Innovations	Effects	References
Natural +hydrogels	Hyaluronic acid	Not specified	Neural tissue regeneration *in vitro*	Sustained delivery of neurotrophin-3	Enhanced neural tissue regeneration	Ramos Ferrer et al. (2024)
	Gelatin, Hyaluronic acid	Not specified	Bone regeneration (3D printed titanium cage)	Hybrid hydrogels for bone tissue engineering	Gelatin/hyaluronic acid-based hydrogel bridges enhance bone regeneration	Park et al. (2023)
	Hyaluronan, Gelatin	Not specified	Spinal cord injury treatment in rats	Encapsulation of spinal cord progenitor cells in hydrogels	Enhanced spinal cord repair and regeneration	Kwokdinata et al. (2023)
	Silk, Collagen	Mechano-tuned crosslinking	Spinal cord injury in rats	Mechano-tuned silk-collagen hydrogels for neural stem cell delivery	Enhanced recovery of contused spinal cord in rats	Davaa et al. (2023)
	Silk fibroin	Not specified	Spinal cord injury treatment in rats	Bioactive, adhesive silk fibroin hydrogel for spinal cord injury treatment	Enhanced tissue repair and bioactivity	Liu et al. (2023a)
Composite hydrogels	Hyaluronic acid, silk fibroin, polydopamine, NT-3	Physical and chemical crosslinking	SCI rat model	Biomimetic scaffold with NT-3 release	Promotes neuronal survival and axonal regeneration	Sha et al. (2023)
	Sodium alginate, silicon nitride, PVA	Ionic crosslinking	Not specified	Improved damping properties for spinal cord applications	Enhances mechanical stability and adaptability	Du et al. (2024)
	Gelatin methacrylate, nerve growth factors	UV-induced crosslinking	SCI rat model	Supports neural stem cell proliferation and differentiation	Enhances neural regeneration and functional recovery	Shen et al. (2024)
	Star-shaped PEG, glycosaminoglycan, adipose-derived stem cell secretome	Covalent crosslinking	Complete transection SCI rat model	Sustained release of stem cell secretome	Improves motor recovery after SCI	Silva et al. (2023)
	Gelatin, sodium alginate, PLGA-curcumin nanoparticles, human endometrial stem cells	Ionic and covalent crosslinking	SCI rat model	Combination of stem cell therapy and antioxidant-loaded nanoparticles	Enhances neuroprotection and nerve regeneration	Ai et al. (2023)

#### 2.1.1 Natural hydrogels

Natural hydrogels are primarily composed of biologically sourced polymers, such as proteins (e.g., collagen, gelatin, fibrin) and polysaccharides (e.g., hyaluronic acid, chitosan, alginate). These hydrogels exhibit excellent biocompatibility and biodegradability, allowing them to mimic the ECM and provide ideal support for the adhesion, growth, and differentiation of neural cells. Additionally, some natural hydrogels also possess the ability to promote neuroprotection, anti-inflammatory effects, and neural regeneration (Najafi et al., 2024; Jia et al., 2023).

For instance, Ramos et al. utilized hyaluronic acid hydrogels as a delivery vehicle for the neurotrophic factor neurotrophin-3 (NT-3). By controlling the degradation rate of the hydrogel, they achieved sustained release of NT-3, which promoted neuronal survival and enhanced the remodeling of neural networks (Ramos Ferrer et al., 2024). Park et al. and Kwokdinata et al. combined gelatin and hyaluronic acid to create bioactive hydrogels. Hyaluronic acid imparted excellent water retention and cell migration properties, while gelatin provided strong cell adhesion and biodegradability. This composite hydrogel was employed in 3D-printed titanium scaffolds for bone regeneration and for encapsulating and delivering spinal cord progenitor cells in spinal cord injury repair, improving cell survival rates and promoting neural regeneration (Park et al., 2023; Kwokdinata et al., 2023). Davaa et al. developed hydrogels with tunable mechanical properties based on silk fibroin and collagen. By adjusting the collagen ratio, they optimized the mechanical strength of the hydrogel to more closely match the biomechanical environment of spinal cord tissue. These hydrogels were used for delivering induced neural stem cells (iNSCs), resulting in improved iNSC survival and promoting axon growth and functional recovery (Davaa et al., 2023). Liu et al. developed a silk fibroin hydrogel with enhanced tissue adhesion via chemical modifications, allowing it to securely adhere to the injury site and form a stable repair microenvironment. Moreover, this hydrogel exhibited excellent bioactivity, promoting neuronal adhesion and axonal growth (Liu et al., 2023a).

However, natural hydrogels often suffer from low mechanical strength and poorly controlled degradation rates, which limit their effectiveness in applications such as drug delivery and cell transplantation. As a result, in SCI repair, pure natural hydrogels typically require further modification or combination with other materials to enhance their stability and functional performance.

#### 2.1.2 Composite hydrogels

Composite hydrogels are typically formed by integrating natural hydrogels with synthetic polymers or incorporating functional nanomaterials into the hydrogel matrix to enhance their mechanical properties, bioactivity, and neuro-regenerative potential. Since purely synthetic hydrogels are rarely used alone, it is essential to briefly introduce their fundamental characteristics before discussing composite hydrogels. Synthetic hydrogels are primarily composed of artificially synthesized polymers such as polyethylene glycol (PEG), poly (lactic-co-glycolic acid) (PLGA), and polyacrylamide (PAM) (Rybachuk et al., 2023). These hydrogels exhibit excellent chemical stability and mechanical strength, with tunable degradation rates, making them suitable for controlled drug release and providing structural support for neural tissue growth. However, due to their lack of bioactivity, synthetic hydrogels have limited interactions with cells and tissues, and their degradation process may generate acidic byproducts, which could hinder neural repair. To overcome these limitations, synthetic hydrogels in SCI treatment are often combined with natural polymers, bioactive molecules, or nanomaterials to improve their biological performance (Gao et al., 2024).

Composite hydrogels integrate the bioactivity of natural hydrogels with the tunable mechanical properties of synthetic hydrogels, making them highly promising for SCI repair. Sha et al. developed a hyaluronic acid/silk fibroin/polydopamine (HA/SF/PDA) composite hydrogel scaffold loaded with the NT-3. This scaffold not only provided stable physical support but also promoted neuronal survival and axonal extension (Sha et al., 2023). Du et al. designed a sodium alginate-silicon nitride-polyvinyl alcohol (SA-Si3N4-PVA) composite hydrogel, optimizing its damping properties to enhance mechanical stability, making it more suitable for complex physiological environments (Du et al., 2024). Shen et al. utilized a gelatin methacryloyl (GelMA) hydrogel incorporating nerve growth factor (NGF), which significantly enhanced neural stem cell proliferation and differentiation, thereby accelerating SCI repair (Shen et al., 2024). Silva et al. employed a star-shaped polyethylene glycol-glycosaminoglycan (PEG-GAG) composite hydrogel as a controlled release carrier for the secretome of human adipose-derived stem cells. In a rat model of complete spinal cord transection, this hydrogel facilitated motor function recovery (Silva et al., 2023). Ai et al. developed a gelatin/sodium alginate (Gel/SA) composite hydrogel scaffold loaded with curcumin-containing PLGA nanoparticles and human endometrial stem cells (hEnSCs). This design not only enhanced the hydrogel’s antioxidant properties but also improved neural regeneration efficiency (Ai et al., 2023). In summary, composite hydrogels leverage the advantages of both synthetic and natural polymers, offering superior mechanical performance, biocompatibility, and neuro-regenerative potential.

### 2.2 Hydrogels categorized by morphology

**FIGURE 2:**
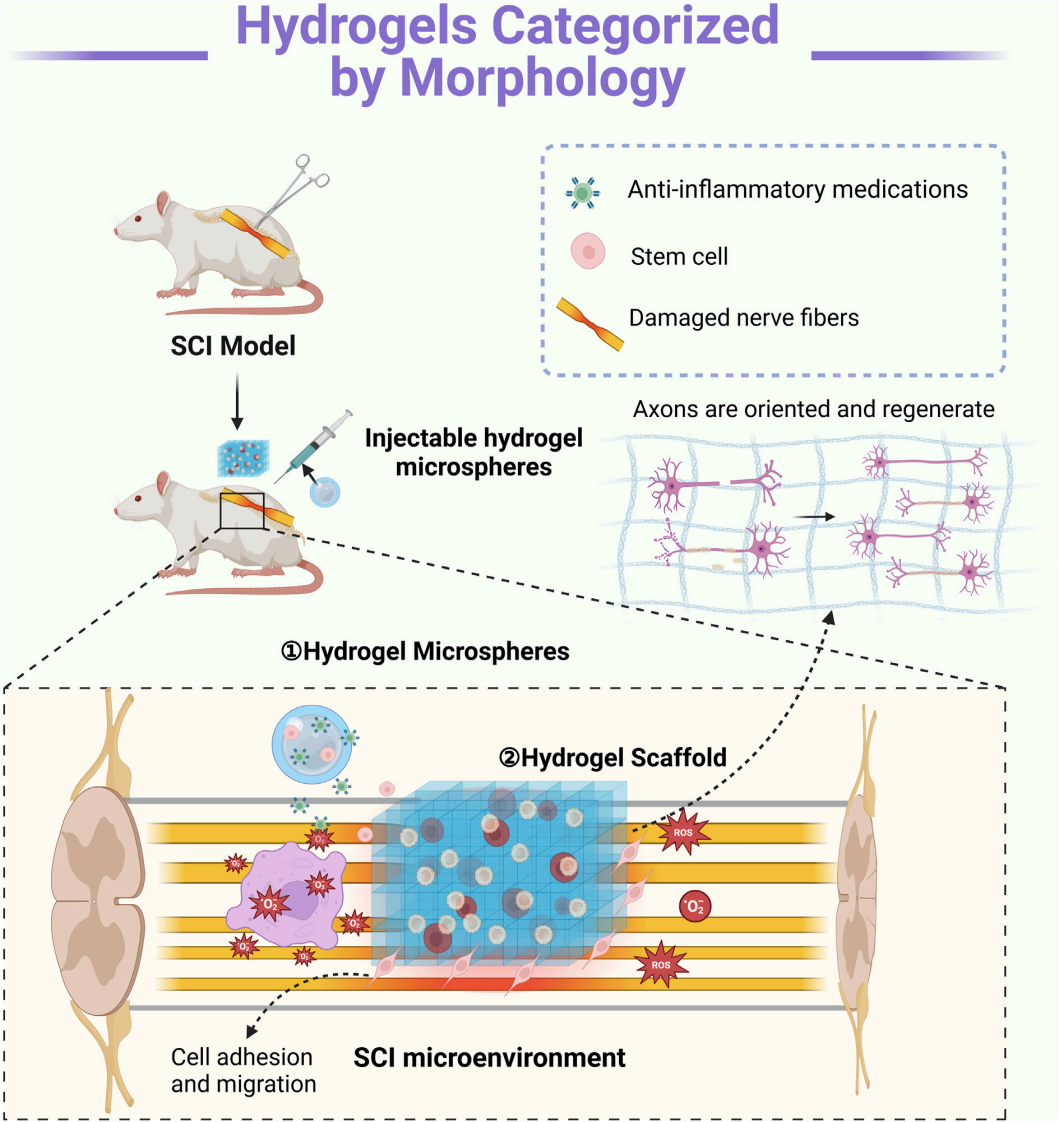
Mechanisms of Action of Hydrogels with Different Morphologies: ① Hydrogel microspheres enable precise local delivery, serving as carriers for drugs and growth factors. These bioactive agents are released gradually into the injury site, modulating inflammation and promoting nerve regeneration. ② Hydrogel scaffolds provide structural support and guidance by mimicking the extracellular matrix in three dimensions. They facilitate axonal regrowth by directing neurite extension while supporting cell adhesion and migration. Typically, these scaffolds require surgical implantation (Created in https://BioRender.com).

In the context of SCI rehabilitation, hydrogels can be categorized by morphology into hydrogel microspheres and hydrogel scaffolds, each offering distinct advantages. Hydrogel microspheres provide a high surface-area-to-volume ratio, facilitating cell adhesion, drug delivery, and controlled release of bioactive molecules (Bobis-Wozowicz et al., 2015). Hydrogel scaffolds serve as three-dimensional frameworks that mimic the ECM, promoting cell proliferation, axonal regeneration, and tissue remodeling (Roh et al., 2023). This morphological classification underscores the versatility of hydrogels in addressing different aspects of SCI repair and functional recovery (Table 2) (Figure 2).

**TABLE 2:** Hydrogels categorized by morphology.

Classification	Composition	Crosslinking method	Preparation technology	Biological model	Innovations	Effects	References
Hydrogel Microspheres	Platelet-Derived Growth Factor Mimetic Peptide (PDGF-mimetic peptide)	Physical crosslinking (Supramolecular interactions)	Synthesis of supramolecular hydrogel microspheres, addition of mimetic peptides for repair	SCI mouse model	Utilization of supramolecular self-assembly combined with PDGF mimetic peptide for neural repair	Improves spinal cord injury repair, promotes neural recovery, and reduces inflammation	Wu et al. (2023)
	Tetrandrine nanoparticles, microgels (microcapsules)	Physical crosslinking (Combination of microgel and nanoparticles)	Microgel encapsulation of tetrandrine nanoparticles to sustain neuroinflammation inhibition	Rat SCI model	Microgel encapsulation of nanoparticles, controlled drug release, and inhibition of neuroinflammation	Enhanced spinal cord repair, inhibition of neuroinflammation, and promotion of neural function recovery	Xu et al. (2025)
	Chitosan, Zinc-Doped Bioactive Glasses, Hydrogel Microspheres	Physical crosslinking (Chitosan modification and hydrogel crosslinking)	Preparation of chitosan-modified hydrogel microspheres, encapsulation of zinc-doped bioactive glasses	Mouse SCI model	Chitosan-modified hydrogel microspheres encapsulating zinc-doped bioactive glass to promote angiogenesis and suppress inflammation	Promotes angiogenesis, suppresses inflammation, and improves neural repair	Su et al. (2025)
	CNS organoids, microgel scaffold	No crosslinking (Organoids formed by cell culture and self-assembly)	Creation of organoid model, surpassing cell-laden microgel scaffolds in repair efficacy	Mouse SCI model	Self-assembly of CNS organoids surpassing microgel scaffolds for better repair outcomes	Promotes spinal cord injury repair, improves functional recovery, and outperforms cell-laden microgel scaffolds	Wang et al. (2022)
Hydrogel Scaffold	MG53 protein, GMs, HA-Dex	Chemical crosslinking of proteins and polysaccharides	Composite hydrogel formation using MG53, GMs, and HA-Dex	*In vitro* neuronal cells, SCI model	Combination of neuroprotective protein, controlled degradation of microspheres, ECM-mimetic HA-Dex matrix	Reduced neuroinflammation, functional recovery in SCI models	Li et al. (2024a)
	Bioactive hydrogel with tunable mechanical properties	Physical and chemical crosslinking	Crosslinking using bioactive agents for mechanical tuning	Rat SCI model	Study of biomechanical properties on neural repair outcomes	Enhanced neural repair, reduced inflammation with soft scaffolds	Zheng et al. (2025)
	3D-printed hydrogel with optimized microstructure	3D printing (precise control over structure and porosity)	3D printing for microstructure control	Rat SCI model	3D printing technology to create custom microstructures for better neuronal interaction	Improved neuronal attachment and axonal growth, better tissue repair	Zhang et al. (2021)
	TiO_2_ self-assembled monolayer (phosphonates)	Physisorption-based surface modification (TiO_2_ layer)	Surface modification with TiO_2_ monolayers	*In vitro* neuronal survival assays, SCI models	Surface functionalization with TiO_2_ for oxidative stress reduction	Increased neuronal survival, reduced oxidative stress, optimized SCI repair	Siddiqui et al. (2023)
	Clickable hydrogel for cell delivery	Click chemistry for particle crosslinking	Clickable chemistry for stable scaffold formation	SCI models	Clickable hydrogels to improve cell delivery and microenvironment	Efficient NPC delivery, improved repair microenvironment	Tigner et al. (2024)

#### 2.2.1 Hydrogel microspheres

Hydrogel microspheres are three-dimensional (3D) micro-structured hydrogels characterized by controllable size, high surface area, excellent fluidity, and injectability. Their micron-scale dimensions enable efficient tissue penetration, making them highly adaptable to complex injury microenvironments (Zheng et al., 2024). By serving as carriers for bioactive factors or cells, hydrogel microspheres play a crucial role in promoting SCI repair, offering several key advantages over bulk hydrogels, including more uniform biological distribution for enhanced drug or growth factor delivery, precise shape control for targeted injection through fluid dynamics, and a large surface area with a tunable degradation rate that facilitates cellular interactions and tissue regeneration (Zhang et al., 2021; Nazerian et al., 2023).

In terms of growth factor delivery, Wu et al. developed hydrogel microspheres using a supramolecular self-assembly strategy to encapsulate platelet-derived growth factor-mimetic peptides (PDGF-MP). This design effectively promoted neuro-regeneration and angiogenesis while providing sustained bioactive signaling for a more stable repair environment. Beyond growth factor delivery, hydrogel microspheres also serve as effective carriers for controlled small-molecule drug release, which can modulate inflammatory responses following SCI (Wu et al., 2023). For instance, Xu et al. synthesized hydrogel microspheres via emulsion cross-linking polymerization, encapsulating tetrandrine (TET) nanoparticles to achieve prolonged inhibition of neuroinflammation while minimizing systemic side effects. To further enhance their bioactivity, researchers have combined hydrogel microspheres with other bioactive materials (Xu et al., 2025). Su et al. employed an ionic cross-linking method to modify hydrogel microspheres with chitosan and integrate them with zinc-doped bioactive glass (Zn-BGs). This composite system not only provided anti-inflammatory effects but also promoted vascularization, highlighting its potential for microenvironment modulation (Su et al., 2025). In addition, Wang et al. developed a cell-laden hydrogel microsphere system using microencapsulation technology, enabling the assembly of CNS organoid-like structures to facilitate neural network reconstruction, offering a novel cell therapy approach for SCI repair (Wang et al., 2022).

#### 2.2.2 Hydrogel scaffold

Hydrogel scaffolds are three-dimensional, porous, solid hydrogel structures designed to fill SCI cavities, providing physical support while guiding axonal regeneration (Cai et al., 2023). Compared to hydrogel microspheres and injectable hydrogels, hydrogel scaffolds offer a more stable macroscopic framework, ensuring sustained structural integrity at the injury site and preventing tissue collapse (Roh et al., 2023). Their interconnected network mimics the ECM more effectively than the discrete nature of microspheres or the fluidity of injectable hydrogels, thereby promoting cell adhesion, neurite extension, and vascularization. Additionally, the mechanical properties and degradation rate of hydrogel scaffolds can be finely tuned to match different stages of tissue repair (Santos et al., 2024). They also serve as carriers for bioactive molecules or cells, enhancing neural regeneration. In recent years, researchers have developed innovative strategies to enhance hydrogel scaffolds by optimizing their mechanical properties, incorporating bioactive modifications, and refining cell delivery techniques (Zhang et al., 2024a).

For example, Li et al. designed a composite hydrogel scaffold incorporating MG53 protein, gelatin microspheres (GMs), and a hyaluronic acid-dextran (HA-Dex) matrix. This combination leverages MG53’s neuroprotective effects, GMs’ controlled degradation, and HA-Dex’s reparative properties to mitigate neuroinflammation and promote functional recovery. Beyond bioactivity, the mechanical properties of hydrogel scaffolds play a pivotal role in SCI repair (Li et al., 2024a). Zheng et al. demonstrated that softer scaffolds reduce inflammation and enhance neural repair, whereas stiffer scaffolds may exacerbate inflammation and hinder tissue regeneration. This highlights the crucial role of biomechanical cues in directing SCI recovery. Meanwhile, advances in fabrication techniques have further refined scaffold designs (Zheng et al., 2025). Zhang et al. utilized 3D printing to precisely control scaffold microarchitecture and porosity, thereby better mimicking the ECM and facilitating neuronal attachment and axonal growth. This approach also enhances scaffold degradability and adaptability, making it more responsive to the dynamic needs of tissue repair. Surface modifications have also emerged as a promising strategy for enhancing scaffold performance (Khaledian et al., 2024; Zhang et al., 202b). Siddiqui et al. introduced a titanium dioxide (TiO_2_) self-assembled monolayer of phosphonates onto the scaffold surface, which improved neuronal survival and reduced oxidative stress, thereby creating a more favorable microenvironment for SCI recovery. Furthermore, novel cell delivery methods have expanded the therapeutic potential of hydrogel scaffolds (Siddiqui et al., 2023). Tigner et al. developed clickable granular hydrogel scaffolds that use click chemistry to form stable structures, enabling the efficient delivery of neural progenitor cells (NPCs) while simultaneously optimizing the injury microenvironment. This strategy offers a promising approach for cell-based therapies in SCI treatment (Tigner et al., 2024).

### 2.3 Hydrogels categorized by functional properties

#### 2.3.1 Stimulus-responsive hydrogels

Stimuli-responsive hydrogels are a class of smart materials capable of undergoing reversible changes in shape or function when exposed to external physical, chemical, or biological stimuli. These hydrogels offer distinct advantages in SCI repair (Sun et al., 2023). Typically, their response mechanisms rely on specific molecules or nanoparticles within the crosslinked network, which undergo structural or bond-energy changes when subjected to particular stimuli, thereby inducing hydrogel swelling, degradation, or the release of bioactive factors (Xiao et al., 2023) (Table 3).

**TABLE 3:** Hydrogels categorized by functional properties.

Classification	Composition	Conductivity or cross-linking method	Preparation technology	Biological model	Innovations	Effect and results	References
Stimulus-responsive Hydrogels	PNIPAM, H_2_S release system	Temperature-responsive	Injectable hydrogel	SCI rat model	H_2_S system enhances cell survival	Reduces inflammation, promotes repair	Albashari et al. (2024)
	Photocrosslinkable groups, stem cells	Photo-crosslinking	Co-culture hydrogel	SCI mouse model	Hydrogel supports stem cell co-culture	Promotes neural repair	Bai et al. (2024)
	Bisphosphonates, minocycline	pH-responsive	Drug-loaded hydrogel	SCI mouse model	pH-controlled drug release	Reduces inflammation, regulates macrophages	Li et al. (2024b)
	Hyaluronidase, magnetic nanoparticles	Magnetic field-induced	Magnetic hydrogel	SCI mouse model	Magnetic field guides axon growth	Enhances regeneration	Fan et al. (2025)
	Thioether/selenoether polymers	ROS-responsive	Redox-controlled hydrogel	SCI rat model	ROS response controls degradation	Supports cell growth, repair	Chen et al. (2025)
	ZnMn@SF nanocomposite	Metal ion crosslinking	Ion-controlled hydrogel	SCI mouse model	Metal ions regulate macrophages	Promotes neural repair	Cui et al. (2024)
Conductive hydrogels	Polyaniline	∼10 S/m	Micro-nanostructured assembly	Rat model, 1 Hz, 50 μA	Enhances nerve regeneration via electrical stimulation	Enhanced axonal growth and nerve regeneration	Xu et al. (2025)
	PEDOT	∼1.5 S/m	Injectable, neuroelectric signal mimicking	Rat model, 10 Hz, 100 μA	Mimics neuroelectric signals, modulates microenvironment	Improved functional recovery, reduced inflammation	Ye et al. (2024)
	PEDOT, Donepezil	∼5 S/m	Self-healing, stem cell and drug delivery	Rat model, 5 Hz, 50 μA	Self-healing, enhances local therapy with stem cells	Enhanced axonal regeneration, improved motor function	Liu et al. (2023b)
	Conductive polymer (unspecified)	∼2 S/m	Bioactive molecule delivery	Rat model, 20 Hz, 200 μA	Restores electrical conduction, supports nerve regeneration	Improved neurological recovery	Zhang et al. (2021)
	Silk, PEDOT:PSS	∼3 S/m	Silk-based, electrically conductive	Rat and mouse models,10 Hz, 100 μA	Silk/PEDOT:PSS hybrid, promotes neural network formation	Improved neural network connectivity, enhanced regeneration	Borah et al. (2025)
Directional fiber hydrogel	Alginate	Ionic cross-linking with calcium chloride	Extrusion	*In vitro* neural cell culture	Mimics neural alignment, enhances neurite outgrowth	Oriented microfibers enhance alignment and neurite outgrowth	Ghaderinejad et al. (2021)
	Collagen, PEG	Covalent cross-linking using PEG	Electrospinning	*In vitro* neural stem cell culture	Replicates neural topography, improves neural differentiation	Biomimetic aligned fibers improve differentiation and elongation	Severs et al. (2024)
Self-healing hydrogel	Curcumin-loaded nanoparticles	Dynamic Schiff base reactions, ionic cross-linking	Nanoparticle incorporation	Rat SCI model	Self-healing, sustained curcumin release, anti-inflammatory	Reduced stress, inflammation; improved regeneration, motor recovery	Luo et al. (2021)
	Gelatin methacrylate, conductive polymers	Dynamic covalent bonds	Composite polymer synthesis	Rat SCI model	Self-healing, electroconductive, neural repair microenvironment	Enhanced neural repair, reduced inflammation, functional recovery	Luo et al. (2022)
	Tauroursodeoxycholic acid (TUDCA)	Dynamic covalent cross-linking	Dynamic polymerization	Rat SCI model	Self-healing, anti-inflammatory, tissue protection	Reduced inflammation, promoted axonal regrowth, motor improvement	Han et al. (2020)

**FIGURE 3:**
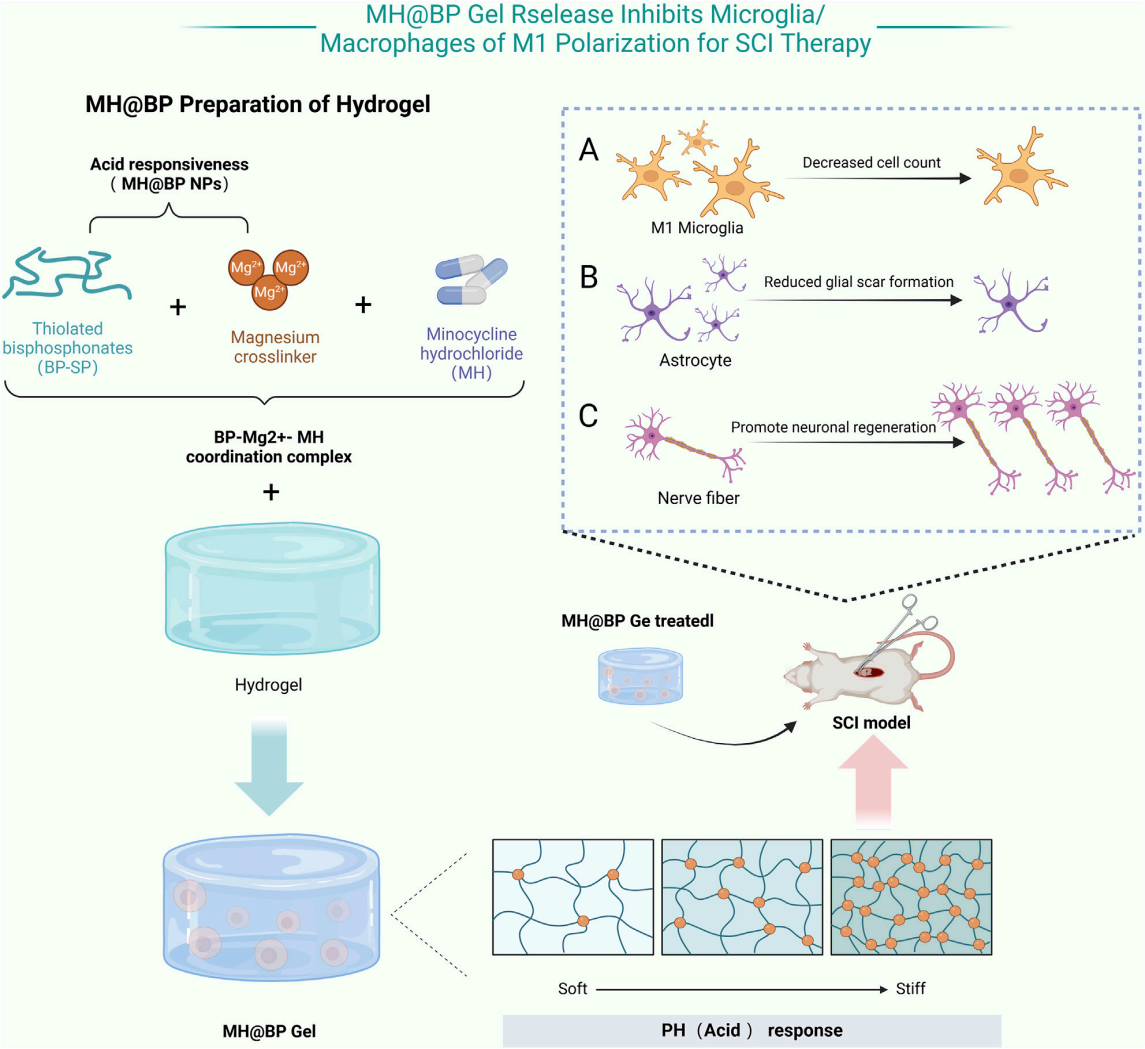
MH@BP pH-Responsive Hydrogel: This hydrogel consisting of dextran sulfate, MH, and magnesium ions (Mg^2+^), utilizes acid-responsive nanoparticles (NPs) based on bisphosphonates (BP-SH) and Mg^2+^ formed through coordination bonds. Minocycline hydrochloride (MH) selectively inhibits the polarization of microglia toward the M1 phenotype without affecting the expression levels of M2 microglial markers. When applied locally for SCI treatment, this composite hydrogel effectively attenuates the inflammatory response induced by M1 microglia, reduces scar formation, and promotes neuronal repair in the SCI (Created in https://BioRender.com).

Li et al. developed a pH-responsive hydrogel incorporating bisphosphonate and minocycline, designed to degrade in acidic environments. At the injury site, where the local microenvironment tends to be acidic, bisphosphonate undergoes degradation, gradually releasing minocycline. This controlled release helps suppress the activation of M1 microglia and macrophages, thereby reducing the secretion of pro-inflammatory cytokines. As a result, inflammation is effectively mitigated, the microenvironment for tissue repair is optimized, and neuronal regeneration is promoted (Li et al., 2024b) (Figure 3). Similarly, Albashari et al. designed a thermosensitive hydrogel based on poly (N-isopropylacrylamide) (PNIPAM). This hydrogel remains in a liquid state at lower temperatures but rapidly transitions into a gel at physiological temperature (37°C), forming an injectable three-dimensional scaffold. Additionally, it continuously releases hydrogen sulfide (H_2_S), which enhances endogenous stem cell survival and reduces secondary injury through its antioxidative and anti-inflammatory properties, further facilitating neural tissue repair (Albashari et al., 2024). In the realm of functional hydrogels, Bai et al. developed a light-responsive hydrogel incorporating photocrosslinkable groups such as azobenzene or photosensitive proteins. When exposed to specific wavelengths of light, these hydrogels undergo conformational changes or free-radical crosslinking, improving their mechanical stability. This process also enhances the adhesion, proliferation, and differentiation of neural stem cells, creating a more favorable microenvironment for nerve regeneration (Bai et al., 2024). Meanwhile, Fan et al. engineered a magnetically responsive hydrogel that integrates hyaluronidase for controlled degradation and magnetic nanoparticles that respond to an external magnetic field. The applied magnetic force induces microscopic reorganization of the hydrogel network, guiding axonal growth and applying localized mechanical stimulation. This approach not only improves the microenvironment at the injury site but also enhances the overall efficacy of tissue repair (Fan et al., 2025; Tran et al., 2022).

Beyond these functional hydrogels, Chen et al. designed a reactive oxygen species (ROS)-responsive hydrogel composed of oxidatively degradable thioether or selenoether polymers. When exposed to elevated ROS levels in the injury region, the hydrogel matrix undergoes oxidative degradation, simultaneously releasing anti-inflammatory agents. This dual-function mechanism alleviates oxidative stress and minimizes secondary inflammatory responses, providing precise microenvironmental modulation for SCI repair (Chen et al., 2025; Jaffer et al., 2023). Cui et al. further expanded the scope of hydrogel-based therapies by developing a metalloprotein-regulating hydrogel incorporating ZnMn@SF nanocomposites. This system enables the controlled release of Zn^2+^ and Mn^2+^, which modulate macrophage-associated metalloprotein expression, shifting M1 pro-inflammatory macrophages toward the M2 anti-inflammatory phenotype. Additionally, these metal ion signals contribute to neuronal survival and axonal extension, further enhancing tissue repair after SCI (Cui et al., 2024).

#### 2.3.2 Conductive hydrogels

Conductive hydrogels are a prominent type of functionalized hydrogel, distinguished by their ability to restore electrical conductivity and support neural regeneration (Shahemi et al., 2023). Commonly used conductive materials for SCI repair include polyaniline (PANI) (Qin et al., 2023; Zare et al., 2020), polypyrrole (PPy), metallic nanoparticles such as gold and silver, and carbon-based materials like carbon nanotubes (CNT), graphene, and MXenes (Xu et al., 2016) (Table 3).

Xu et al. employed interfacial polymerization to assemble micro-nanostructured polyaniline into a cellulose-based hydrogel, which promoted nerve regeneration by enhancing axonal growth, as verified through electrical stimulation and *in vitro* tests for spinal cord repair (Xu et al., 2016). PEDOT hydrogels are highly conductive, biocompatible, and capable of mimicking neuro-electric signals to facilitate neural regeneration. Their advantages include enhancing neural network formation, promoting axonal growth, reducing inflammation, and improving functional recovery in SCI models (Borah et al., 2025). Ye et al. developed an injectable hydrogel incorporating PEDOT to mimic neuro-electric signals and modulate the injury micro-environment. *In vivo* studies demonstrated improved functional recovery and reduced inflammation in SCI models using electrophysiological assessments and behavioral tests (Ye et al., 2024). Similarly, Liu et al. designed a self-healing hydrogel integrating PEDOT and donepezil, capable of delivering neural stem cells and drugs. This hydrogel enhanced axonal regeneration and motor function in SCI models, as evidenced by *in vivo* imaging and histological analysis (Liu et al., 2023b). Furthermore, Zhang et al. created a crosslinked hydrogel incorporating conductive polymers to restore electrical conduction. Electrophysiological studies and functional recovery tests showed improved neurological recovery in rat SCI models (Zhang et al., 202c).

#### 2.3.3 Oriented fiber and self-repairing hydrogels

Directional fibers and self-healing hydrogels represent innovative biomaterials for SCI repair, offering distinct advantages over traditional hydrogels in structural alignment and dynamic adaptability (Yin et al., 2024). Directional fibers replicate the anisotropic properties of neural tissues, providing physical guidance for axonal regeneration (Rossi et al., 2024), while self-healing hydrogels autonomously recover from mechanical damage and sustain therapeutic delivery (Tang et al., 2023). Together, these materials enhance biocompatibility, cellular interactions, and mechanical stability (Mozhdehbakhsh Mofrad and Shamloo, 2023) (Table 3).

Various anisotropic hydrogels have been developed to enhance SCI repair by replicating the aligned architecture of neural tissues. Ghaderinejad et al. designed an injectable alginate hydrogel with oriented microfibers using extrusion and ionic cross-linking with calcium chloride, which demonstrated enhanced neurite outgrowth *in vitro* along the fiber alignment (Ghaderinejad et al., 2021). Zheng et al. created anisotropic alginate hydrogels through directional freeze-drying followed by ionic cross-linking, producing scaffolds with aligned porous structures that promoted axonal regrowth and reduced inflammation, as validated in a rat SCI model (Zheng et al., 2025). Similarly, Severs et al. constructed biomimetic 3D hydrogels with aligned topography via electrospinning and cross-linking collagen with polyethylene glycol, showing significant neurite elongation and alignment in neural stem cell differentiation assays (Severs et al., 2024). These anisotropic hydrogels not only provide structural guidance for axonal regeneration but also establish a microenvironment conducive to neural repair, offering great potential for advancing SCI treatment.

Several self-healing hydrogels have been developed to enhance SCI repair by creating a dynamic and supportive environment. Luo et al. designed an injectable hydrogel incorporating curcumin-loaded nanoparticles via dynamic Schiff base reactions and ionic cross-linking, achieving sustained curcumin release to reduce oxidative stress and inflammation (Luo et al., 2022). Luo et al. also developed a self-healing, electroconductive hydrogel composed of gelatin methacrylate and conductive polymers cross-linked through dynamic covalent bonds, providing a conductive microenvironment that facilitated neural repair (Luo et al., 2021). Han et al. prepared a hydrogel loaded with tauro-ursodeoxycholic acid using dynamic covalent cross-linking, combining anti-inflammatory effects with tissue-protective properties (Han et al., 2020). In rat SCI models, these hydrogels demonstrated self-repair capacity, reduced inflammation, enhanced axonal regrowth, and improved motor function, as validated through histological, electrophysiological, and behavioral assessments. Collectively, these materials offer a promising platform for neural regeneration and functional recovery.

## 3 Mechanistic roles of hydrogels in SCI

### 3.1 Enhancing neuronal and axonal regeneration

In SCI, primary trauma causes axonal severance and disruption of neural networks, while secondary responses such as inflammation, ischemia, and oxidative stress exacerbate neuronal damage and inhibit axonal regeneration (Sun et al., 2024). Additionally, the formation of glial scars and an ECM enriched with chondroitin sulfate proteoglycans (CSPGs) creates an inhibitory microenvironment post-injury, hindering axonal elongation and neural regeneration (Bajic et al., 2024). Damage to neuronal axons disrupts neural signal conduction, resulting in neurological dysfunctions such as sensory and motor deficits. The lack of efficient axonal regeneration not only impedes spinal cord repair but also accelerates chronic neurological deterioration, severely affecting patients’ quality of life (Zhang et al., 202d).

Hydrogels promote neuronal axonal regeneration in SCI through various mechanisms, with their structural and functional design playing a crucial role in guiding axonal outgrowth. For instance, Zhang et al. developed a conductive hydrogel fiber CNT with GelMA, mimicking axonal alignment. When paired with electrical stimulation, this hydrogel significantly enhanced axonal extension and facilitated spinal cord functional recovery, highlighting the synergistic importance of spatial alignment and conductivity (Zhang et al., 202a). Similarly, Sun Z et al. designed a bioactive and neurotrophic peptide hydrogel with a defined three-dimensional structure and thermosensitive properties, demonstrating remarkable efficacy in promoting directional axonal growth (Sun et al., 2024). Bajic A et al. showed that hyaluronic acid-based hydrogels, optimized via physical and chemical crosslinking, not only improve neuronal adhesion but also substantially enhance axonal elongation, emphasizing the structural importance of hydrogels in neural repair (Bajic et al., 2024).

Hydrogels also act as delivery platforms, providing stable carriers for neuro-regenerative factors (Hlavac et al., 2023). Chemically modified injectable hydrogels enable the targeted delivery of neurotrophic factors such as brain-derived neurotrophic factor (BDNF) and anti-inflammatory agents (Ji et al., 2023; Sorouri et al., 2023), effectively mitigating neuroinflammation, promoting axonal regeneration, and improving the efficacy of neural repair (Meissner et al., 2024a). The injectability and adaptability of hydrogels allow them to seamlessly fill irregularly shaped injury sites, offering precise structural support for neural regeneration (Xie et al., 2025). Recent studies emphasize optimizing hydrogel networks and leveraging synergistic interactions between external stimuli and growth factors to enhance their biological functionalities. For example, Kim et al. utilized Arg-Gly-Asp (RGD)-functionalized hydrogels in combination with preheated neural stem cells, achieving significant improvements in neural repair outcomes and demonstrating the critical role of hydrogels in supporting cellular functions (Kim and Kang, 2024). Wu et al. designed a capacitive-coupling responsive conductive hydrogel scaffold capable of delivering wireless *in vivo* electrical stimulation without external power. This innovation promoted myelinated axonal regeneration and endogenous neural stem cell differentiation, significantly improving neural tissue growth efficiency (Wu et al., 2024).

**FIGURE 4:**
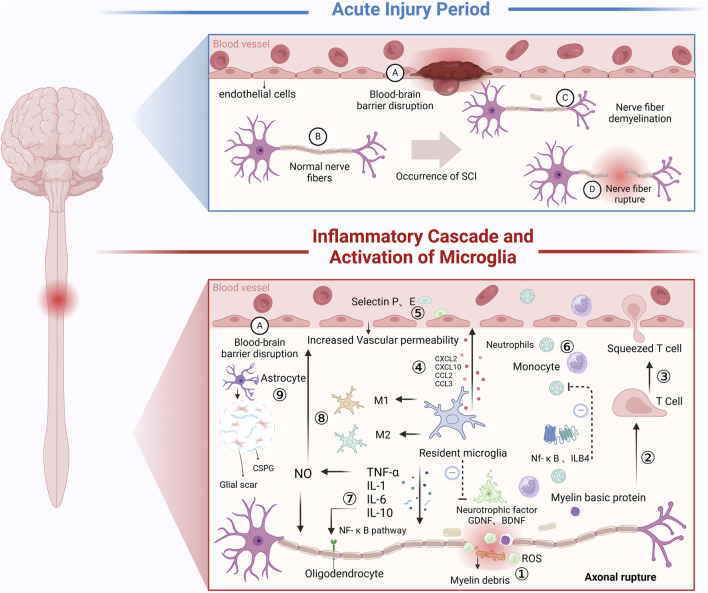
Inflammatory Mechanisms Following SCI: In the acute injury phase: SCI directly causes vascular damage or severing, increasing the permeability of blood vessels. Additionally, neuron damage or severing occurs. After SCI, during the secondary injury phase, a series of inflammatory cascades occur. ① Immune Microenvironment Changes: Local immune microenvironment alterations lead to the release of ROS, which inhibit spinal cord repair. ② Myelin Sheath Disruption: SCI causes myelin sheath rupture, and the myelin basic protein generated in response to demyelination acts as an antigen, inducing T lymphocyte activation. ③ T Lymphocyte Adhesion: T lymphocytes use their cell adhesion molecules to adhere to endothelial cells. ④ Microglia Activation: Resident microglia are rapidly activated following SCI, releasing chemokines (e.g., CXCL2, CXCL10, CCL2, CCL3) and inflammatory mediators (e.g., IL-1, IL-6, TNF-α). Some of these inflammatory mediators create an inhibitory immune microenvironment that suppresses SCI repair. ⑤ Endothelial Cell Activation: The inflammatory response from microglia and T lymphocytes promotes endothelial cells to secrete P-selectin and E-selectin, increasing the permeability of the BSCB. ⑥ Lymphocyte Migration: Due to changes in vascular permeability and the presence of chemokines, lymphocytes are released from blood vessels and migrate to the injury site. Leukotriene B4 (LTB4), a pro-inflammatory leukotriene, and the NF-κB signaling pathway play a role in alleviating neutrophil infiltration, thus contributing to the inflammation in SCI. ⑦ Cell Adhesion Molecules (CAMs): CAMs are cell surface glycoproteins that facilitate cell-cell and cell-tissue interactions. They amplify inflammation and inhibit SCI repair. TNF and interleukins enhance CAM function via the NF-κB pathway. ⑧ TNF and Inflammatory Cascade: Tumor necrosis factor (TNF) enhances NO activity and induces the chemotaxis of inflammatory cells, increasing BSCB permeability and promoting the release of inflammatory cells and mediators, which hinder SCI recovery. ⑨ Astrocyte Activation: Following SCI, astrocytes immediately secrete CSPG, which help form a gliotic scar that acts as a protective barrier, limiting the spread of necrotic and apoptotic cells. However, the persistent presence of CSPG for months hinders cell regeneration and neural repair following SCI (Created in https://BioRender.com).

In conclusion, hydrogels support neuronal axonal regeneration through structural guidance, delivery of bioactive molecules, and responsiveness to external stimuli. These advancements provide a robust theoretical foundation and valuable technical insights for optimizing and applying hydrogels in SCI therapy (Figure 4).

### 3.2 Microenvironment modulation: anti-inflammatory and antioxidative effects

**FIGURE 5:**
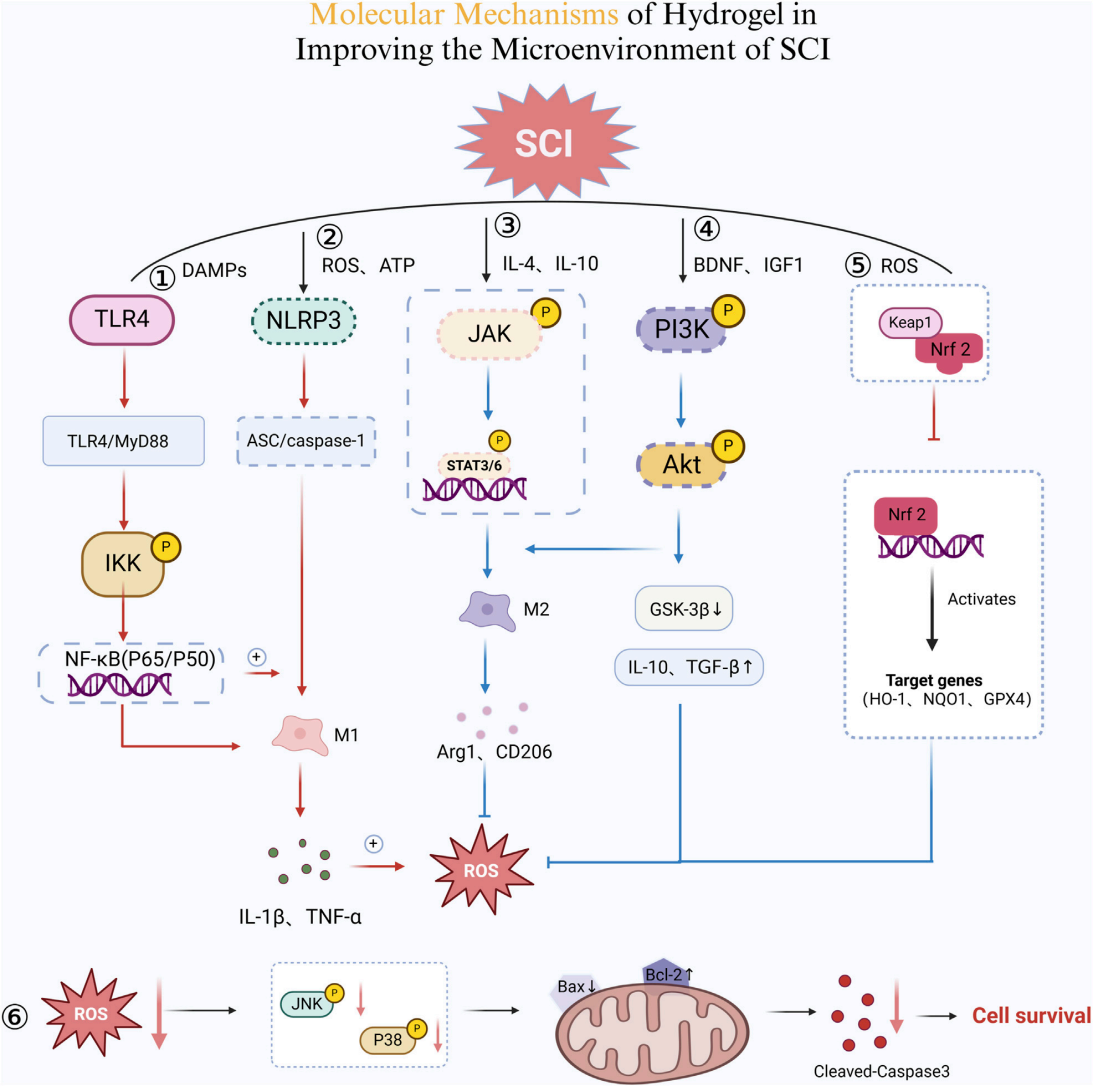
Molecular Mechanisms of Hydrogel in Improving the Microenvironment of SCI. ① TLR4/NF-κB Pro-inflammatory Pathway. Damage-associated molecular patterns (DAMPs) activate TLR4, which triggers the MyD88-dependent pathway. This leads to IKK phosphorylation, promoting NF-κB (P65/P50) nuclear translocation. Consequently, pro-inflammatory cytokines IL-1β and TNF-α are released, sustaining the M1 macrophage phenotype and amplifying inflammation. ② NLRP3 Inflammasome Activation. Excessive ROS and ATP accumulation activates NLRP3, which recruits ASC and caspase-1. This cascade enhances IL-1β and TNF-α secretion, reinforcing M1 macrophage polarization and inflammation. Additionally, a positive feedback loop further elevates ROS levels, exacerbating tissue damage. ③ JAK/STAT Pathway and M2 Macrophage Polarization. IL-4 and IL-10 stimulate JAK phosphorylation, activating STAT3/6. This shift promotes M2 macrophage differentiation, upregulating Arg1 and CD206 while reducing inflammation. ④ PI3K/Akt Pathway for Cell Survival and Repair. BDNF and IGF-1 activate PI3K, leading to Akt phosphorylation. This suppresses GSK-3β and increases IL-10 and TGF-β, enhancing M2 macrophage polarization and neuroprotection. ⑤ Nrf2 Antioxidant Pathway. Reduced oxidative stress promotes Keap1 degradation, freeing Nrf2 to enter the nucleus. Nrf2 induces HO-1, NQO1, and GPX4 expression, neutralizing ROS and mitigating inflammation. ⑥ ROS and Apoptotic Pathway. Lower ROS levels inhibit the JNK/P38 pathway, reducing Bax expression while increasing Bcl-2. This prevents cytochrome c release, ultimately suppressing Caspase-3-mediated apoptosis, promoting cell survival (Created in https://BioRender.com).

Neuroinflammation is one of the primary secondary changes following SCI, which is mainly triggered by the activation of microglia, astrocytes, and associated immune factors such as cytokines and chemokines (Rowland et al., 2008). On the one hand, neuroinflammation plays a beneficial role in the early stages of neural injury by mobilizing immune cells to eliminate pathogens, clear necrotic cells and cellular debris (Freyermuth-Trujillo et al., 2022), and create a favorable environment for CNS repair. Additionally, it promotes neural regeneration by releasing neurotrophic factors such as BDNF (Bronzuoli et al., 2016; Zhang et al., 202b). On the other hand, excessive or prolonged neuroinflammation can severely damage the CNS. For instance, the excessive release of inflammatory cytokines (e.g., IL-1β, TNF-α) can induce neuronal apoptosis and disrupt neural circuits (Kozyrev et al., 2016). Persistent activation of microglia may exacerbate neuronal damage, while neuroinflammation can compromise the integrity of the BBB, leading to excessive infiltration of peripheral immune cells. This further amplifies the inflammatory response, aggravates neuronal damage, and hinders spinal cord recovery after SCI (Glass et al., 2010). Moreover, the production of ROS and free radicals is a consequence of cellular metabolism and external stimuli. Under physiological conditions, ROS play essential roles in signal transduction and immune defense (Zlokovic, 2008) (Figure 5). However, after SCI, excessive ROS generated by immune cells—such as superoxide anion (O_2_^−^), hydrogen peroxide (H_2_O_2_), and hydroxyl radicals (OH•)—act as signaling molecules that activate downstream pathways. These pathways upregulate inflammatory cytokine expression, induce lipid peroxidation, protein oxidation, and DNA damage, and activates inflammatory signaling cascades such as nuclear factor-kappa B (NF-κB). These processes lead to persistent inflammation, ultimately hindering neural repair (Teeling and Perry, 2009).

Hydrogels exert multidimensional effects in regulating the microenvironment of SCI. Their primary functions include the sustained release of anti-inflammatory factors, modulation of immune cell behavior, ROS scavenging, suppression of excessive inflammatory responses, and improvement of redox balance (Zrzavy et al., 2021), thereby creating a favorable environment for neural regeneration (Zorov et al., 2014). For instance, Yuan et al. developed a nanozyme hydrogel that incorporates biomimetic antioxidant nanozymes and loads multiple drugs, including lipoic acid, methylcobalamin and selenium (LA/Me/Se NPs-h). This hydrogel mimics endogenous antioxidant enzymes such as superoxide dismutase (SOD) and catalase (CAT). The SOD-mimetic activity catalyzes the conversion of O_2_^−^ into H_2_O_2_, reducing superoxide anion levels. While the CAT-mimetic activity facilitates the decomposition of H_2_O_2_ into H_2_O and O_2_, thereby preventing secondary oxidative damage (Yuan et al., 2024). Furthermore, the nanozyme hydrogel inhibits Keap1-mediated degradation of Nrf2, promoting Nrf2 nuclear translocation and activating antioxidant-related genes (HO-1, NQO1, GPX4). This enhances neuronal resistance to oxidative stress. Moreover, excessive ROS activate the p38 MAPK/JNK signaling pathway, leading to caspase-3-mediated neuronal apoptosis. The nanozyme hydrogel mitigates p38 MAPK/JNK activity, reducing neuronal death and improving SCI repair outcomes.

As mentioned earlier, neuroinflammation following SCI is predominantly mediated by macrophages and microglia, whose polarization states determine the extent of tissue recovery. Microglia exist in two distinct states: M1 and M2. M1 macrophages secrete pro-inflammatory factors such as TNF-α and IL-6, exacerbating inflammatory damage, whereas M2 macrophages release anti-inflammatory cytokines such as IL-10 and TGF-β, facilitating tissue repair (Sun et al., 2024). Correia et al. developed a chitosan-based hydrogel that optimizes the SCI microenvironment by regulating macrophage polarization, inhibiting pro-inflammatory factor release, and promoting anti-inflammatory cytokine secretion. First, the cationic groups (-NH_3_^+^) of chitosan interact with inflammatory cytokines such as TNF-α and IL-1β, reducing their bioactivity and mitigating inflammatory damage. Second, the chitosan hydrogel upregulates the STAT6 signaling pathway, enhancing the expression of M2-associated genes (Arg-1, CD206, IL-10). Furthermore, by inhibiting NF-κB-mediated inflammatory pathways, the hydrogel decreases the release of IL-6, TNF-α, and iNOS from M1 macrophages, thereby reducing local inflammation. Additionally, in this study, the chitosan hydrogel was loaded with DEX for sustained release at the injury site, further suppressing inflammation, preventing scar formation, and improving neural repair (Correia et al., 2024). Furthermore, Su et al. developed chitosan-modified hydrogel microspheres encapsulating zinc-doped bioactive glass. Their study revealed that Zn^2+^ inhibits caspase-8/FADD-dependent apoptotic signaling, thereby reducing neuronal apoptosis induced by SCI and improving the local microenvironment (Su et al., 2025) (Figure 5).

Mousavi et al. utilized a hydrogel-based system to deliver SCs for SCI repair in rats. Their findings demonstrated that SCs exert neuroprotective effects by suppressing the NLRP3 inflammasome and its downstream pathways. Specifically, SCs reduced the expression of ASC and caspase-1, leading to decreased secretion of IL-1β and TNF-α (Mousavi et al., 2019). Xiao et al. and Sipin et al. designed a hydrogel-based paclitaxel (PTX) delivery system that modulates the PI3K/Akt signaling pathway, enhancing neuronal survival and promoting SCI repair. The sustained release of PTX from the hydrogel enhanced Akt phosphorylation, inhibited GSK-3β activity, and upregulated BDNF and IGF-1 expression. These effects facilitated axonal regeneration while reducing glial scar formation. Additionally, PTX promoted macrophage polarization toward the M2 phenotype, increasing IL-10 and TGF-β levels (Xiao et al., 2020; Sipin et al., 2024). These integrated mechanisms underscore the potential of hydrogel-based therapies in modulating the post-SCI microenvironment. By delivering bioactive molecules and cells, hydrogels can activate multiple anti-inflammatory pathways, optimize regenerative conditions at the injury site, and serve as a promising strategy for spinal cord repair and functional recovery (Shi et al., 2024).

### 3.3 Mitigating scar formation

Scarring is an excessive tissue reparative response to injury stimuli, typically involving the formation of glial and fibrotic scars (Rolls et al., 2009). Scar formation primarily stems from three factors, with the first being the overactivation of glial cells and fibroblasts (Zhu et al., 2024). Following SCI, astrocytes, microglia, and perivascular fibroblast-like cells rapidly accumulate at the injury site, releasing substantial amounts of pro-inflammatory cytokines and ECM molecules (Jones et al., 2003). These actions lead to the development of a barrier-like structure. While such scars provide protection by containing the injury and limiting inflammation spread during the acute phase, they also hinder axonal regeneration and neural functional recovery. The second factor is the prolonged release of pro-inflammatory cytokines (Letko Khait et al., 2025). Persistent inflammation post-SCI, driven by elevated levels of mediators like IL-1β and TNF-α, exacerbates scar formation, perpetuating tissue damage in a vicious cycle. The third contributing factor is the excessive accumulation of ECM components (Tan et al., 2024; Jones et al., 2003). Overproduction of ECM molecules, particularly CSPGs, significantly enhances the inhibitory microenvironment at the injury site, impeding axonal elongation and the re-establishment of functional neural connections. Balancing the protective roles of scarring with its inhibitory effects on regeneration remains a critical challenge in SCI repair research.

Hydrogels exhibit significant potential in suppressing scar formation and promoting axonal regeneration. CSPGs, as critical molecular components of the ECM, are expressed in both developing and adult CNS. Extensive *in vitro* studies have shown that CSPGs can restrict neurite outgrowth and inhibit axonal regeneration following CNS injury. Jiang et al. developed an IKVAV (Ile-Lys-Val-Ala-Val) peptide-loaded hydrogel capable of modulating CSPG degradation and inhibiting glial cell activation, effectively reducing glial scar formation. This hydrogel also curtailed fibroblast migration and activation, minimizing fibrotic scars and enhancing the microenvironment for neuronal axonal regeneration (Jiang et al., 2023). Similarly, Zhu et al. utilized a tissue-adaptive hydrogel to deliver PTX for suppressing excessive glial scar formation while simultaneously releasing basic fibroblast growth factor (bFGF) to stimulate axonal regeneration (Zhu et al., 2024). Tan et al. developed a bioinspired composite hydrogel integrating hyaluronic acid-graft-dopamine (HADA) and a designed peptide, HGF-(RADA)4-DGDRGDS (HRR). This hydrogel significantly enhanced the regenerative microenvironment by regulating ECM composition and deposition at the injury site. It reduced CSPG accumulation, facilitated axonal extension, and supported specific neural connection formation (Tan et al., 2024).

In summary, hydrogels mitigate glial and fibroblast activation and limit the deposition of inhibitory ECM molecules, including CSPGs, effectively preventing scar formation post-SCI. When integrated with drug or gene delivery systems, hydrogels further refine the regenerative microenvironment, enhancing axonal extension and the establishment of specific neural connections. These versatile properties position hydrogels as a comprehensive and promising approach for SCI therapy.

### 3.4 Facilitating angiogenesis

After SCI, primary mechanical trauma and subsequent secondary injury responses result in significant vascular damage. Direct mechanical injury disrupts vascular integrity, causing hemorrhage and localized ischemia, which interrupt oxygen and nutrient delivery to the injury site (Kim and Kang, 2024), This exacerbates neuronal apoptosis and worsens the spinal cord microenvironment. During secondary injury, inflammatory responses, oxidative stress, and the release of pro-inflammatory factors severely impair vascular endothelial cell function, disrupt the BBB, and facilitate peripheral immune cell infiltration (Deng et al., 2024). BBB disruption increases vascular permeability, leading to leakage and amplified inflammation, creating a vicious cycle that hinders angiogenesis. The limited regenerative capacity of newly formed blood vessels impairs nutrient delivery and waste clearance, further obstructing axonal elongation, functional recovery, and overall spinal cord repair (Li et al., 2023).

Hydrogels have emerged as promising tools for regulating the injury microenvironment and creating optimal conditions for vascular regeneration by delivering drugs and bioactive molecules. Conductive hydrogels, for instance, enhance neural signal transmission through their electrical conductivity (Deng et al., 2024). Spinal cord-derived microvascular endothelial cells exhibit strong angiogenic capabilities, and their integration into hydrogel systems significantly enhances vascularization at injury sites, supporting neuronal regeneration and functional recovery (You et al., 2023). For example, Su et al. developed chitosan-modified hydrogels incorporating zinc-doped bioactive glass, which promoted endothelial cell proliferation and migration while effectively suppressing local inflammation to support vascular regeneration (Su et al., 2025). Shen et al. engineered a gelatin methacrylate hydrogel system combined with nerve growth factor (NGF), which facilitated neural stem cell proliferation and differentiation while boosting vascular regeneration (Shen et al., 2024). Liu et al. designed a multifunctional injectable hydrogel incorporating black phosphorus nanosheets and tazarotene, which exhibited dual functionalities: alleviating local inflammation through antioxidative and anti-inflammatory effects, and promoting angiogenesis by upregulating vascular endothelial growth factor (VEGF) expression. Additionally, conductive hydrogels loaded with M2 microglia-derived exosomes stimulated endothelial cell proliferation and migration, leveraging their dual role in promoting neural and vascular repair. This approach significantly enhanced neural recovery and established a robust vascular network, facilitating spinal cord regeneration (Guan et al., 2024).

### 3.5 Preserving the blood-spinal cord barrier

Damage to the BSCB is a hallmark of secondary injury in SCI. Mechanical trauma directly disrupts the barrier’s physical integrity, damaging endothelial cells and the basement membrane. Post-SCI inflammatory responses and oxidative stress exacerbate endothelial cell damage and increase BSCB permeability (Xin et al., 2023). Pro-inflammatory cytokines downregulate tight junction proteins such as ZO-1 and Occludin, further compromising BSCB integrity (Shu et al., 2023). The breakdown of both the BBB and BSCB creates significant challenges for spinal cord functional recovery. Firstly, barrier dysfunction permits the infiltration of peripheral immune cells and toxic molecules into spinal cord tissue, exacerbating local inflammation. Secondly, BSCB damage elevates concentrations of neurotoxic molecules like glutamate, intensifying neuronal injury. It also disrupts the homeostatic balance between neurons and glial cells, further impeding tissue regeneration.

Hydrogels show significant potential for BSCB protection and repair through multifunctional designs that provide structural support and deliver therapeutic agents. These unique properties position hydrogels as effective tools for maintaining and restoring BSCB integrity. Deng et al. developed a conductive hydrogel incorporating tetramethylpyrazine (TMP), which mitigates oxidative stress and pro-inflammatory factors, reduces endothelial damage, stabilizes tight junction protein expression, and promotes tissue repair (Deng et al., 2024). Shu et al. utilized a thermosensitive hydrogel for GPR124 protein delivery, significantly enhancing endothelial cell proliferation and barrier reconstruction, thereby improving barrier functionality after SCI (Shu et al., 2023). Chen et al. designed a decellularized matrix-based hydrogel capable of releasing neurotrophic factor NT-3 and anti-inflammatory agents like curcumin to regulate the injury microenvironment, facilitating BSCB repair and accelerating neural regeneration (Chen et al., 202a). Additionally, Wang et al. introduced an injectable hydrogel employing a multifunctional integration strategy that combines cortical neuron-derived exosomes with decellularized ECM. This system effectively suppresses inflammation while enhancing BSCB repair and regeneration, markedly improving overall tissue repair efficiency (Wang et al., 202b).

## 4 Emerging directions for hydrogel applications

### 4.1 Integration of hydrogels with stem cell therapies

Hydrogels are widely used in SCI repair to support cell transplantation and improve the local microenvironment, thereby promoting neuro regeneration and functional recovery (Subbarayan et al., 2024). Stem cells combined with hydrogels for SCI treatment mainly include mesenchymal stem cells (MSCs), NSCs, induced pluripotent stem cells (iPSCs) (Rybachuk et al., 2023), and SCs. Among them, MSCs are the most extensively studied. Based on their sources, MSCs are categorized into BM-MSCs, adipose-derived MSCs (ADSCs), and umbilical cord-derived MSCs (UC-MSCs) (Jeon et al., 2024). BMSCs possess strong neurogenic differentiation potential and are widely investigated for SCI repair. ADSCs are easily accessible and rich in pro-angiogenic factors, facilitating local microcirculation. UC-MSCs exhibit low immunogenicity and high proliferative capacity, offering advantages in allogeneic transplantation settings (Li et al., 202c) (Table 4).

**TABLE 4:** Applications of stem cells and hydrogels in SCI repair.

Stem cell type	Hydrogel composition	Experimental model	Mechanism of action	References
BMSCs	Graphene-Collagen Hydrogel	Rat SCI model	Inhibiting inflammation (NF-κB suppression), reducing TNF-α/IL-6	Agarwal et al. (2024)
ADSCs	Fibrin Hydrogel	Rat SCI model	Promoting angiogenesis (VEGF/bFGF), enhancing axon regeneration	Chandrababu et al. (2023)
UC-MSCs	Dual-Enzymatically Crosslinked Gelatin Hydrogel	Murine SCI model	Promoting neural differentiation (Wnt/β-catenin activation)	Yao et al. (2020)
NSCs	Photosensitive Hydrogel + BMSCs	Mouse SCI model	Enhancing BDNF secretion, increasing axon regeneration	Bai et al. (2024)
iPSCs	Hyaluronic Acid Hydrogel	Human pluripotent stem cell-derived spinal cord organoid model	Regulating pattern formation, promoting NSC-like cell differentiation	Chen et al. (2024a)
SCs	Exosome-Methylprednisolone Composite Patch	Rat SCI model	Reducing neuronal apoptosis (PI3K/Akt pathway), promoting myelin repair	Zhu et al. (2023)

For example, Agarwal et al. developed a graphene-collagen cryogel loaded with BMSCs. This system inhibited activation of the NF-κB signaling pathway, significantly reducing microglial activation at the injury site. Consequently, levels of pro-inflammatory cytokines such as TNF-α and IL-6 decreased, improving the neuroinflammatory microenvironment after SCI and promoting functional recovery (Agarwal et al., 2024). Chandrababu et al. combined ADSCs with fibrin hydrogels and observed enhanced angiogenesis at the lesion site via secretion of vascular endothelial growth factor (VEGF) and bFGF. This approach further improved neuronal survival and axonal regeneration (Chandrababu et al., 2023). Yao et al. employed a dual-enzyme crosslinked gelatin hydrogel loaded with UC-MSCs. This system activated the Wnt/β-catenin signaling pathway, promoting neuronal differentiation of UC-MSCs and enhancing synaptic plasticity at the injury site, leading to improved motor function in a mouse SCI model (Yao et al., 2020). NSCs can directly differentiate into neurons and glial cells, making them valuable for SCI repair. However, their low survival rate necessitates the use of hydrogels as scaffolds to optimize the local microenvironment. Bai et al. found that co-culturing NSCs with photosensitive hydrogels and BMSCs increased NSC survival by enhancing BDNF secretion. This strategy also promoted axonal growth and synapse formation, improving functional recovery after SCI (Bai et al., 2024). Kim et al. used heat-shock-preconditioned NSCs combined with RGD-functionalized hydrogels. This approach upregulated heat shock protein 70 (HSP70), enhancing NSC survival and neuronal differentiation. The RGD motif also improved NSC adhesion, accelerating tissue remodeling at the lesion site (Kim and Kang, 2024).

**FIGURE 6:**
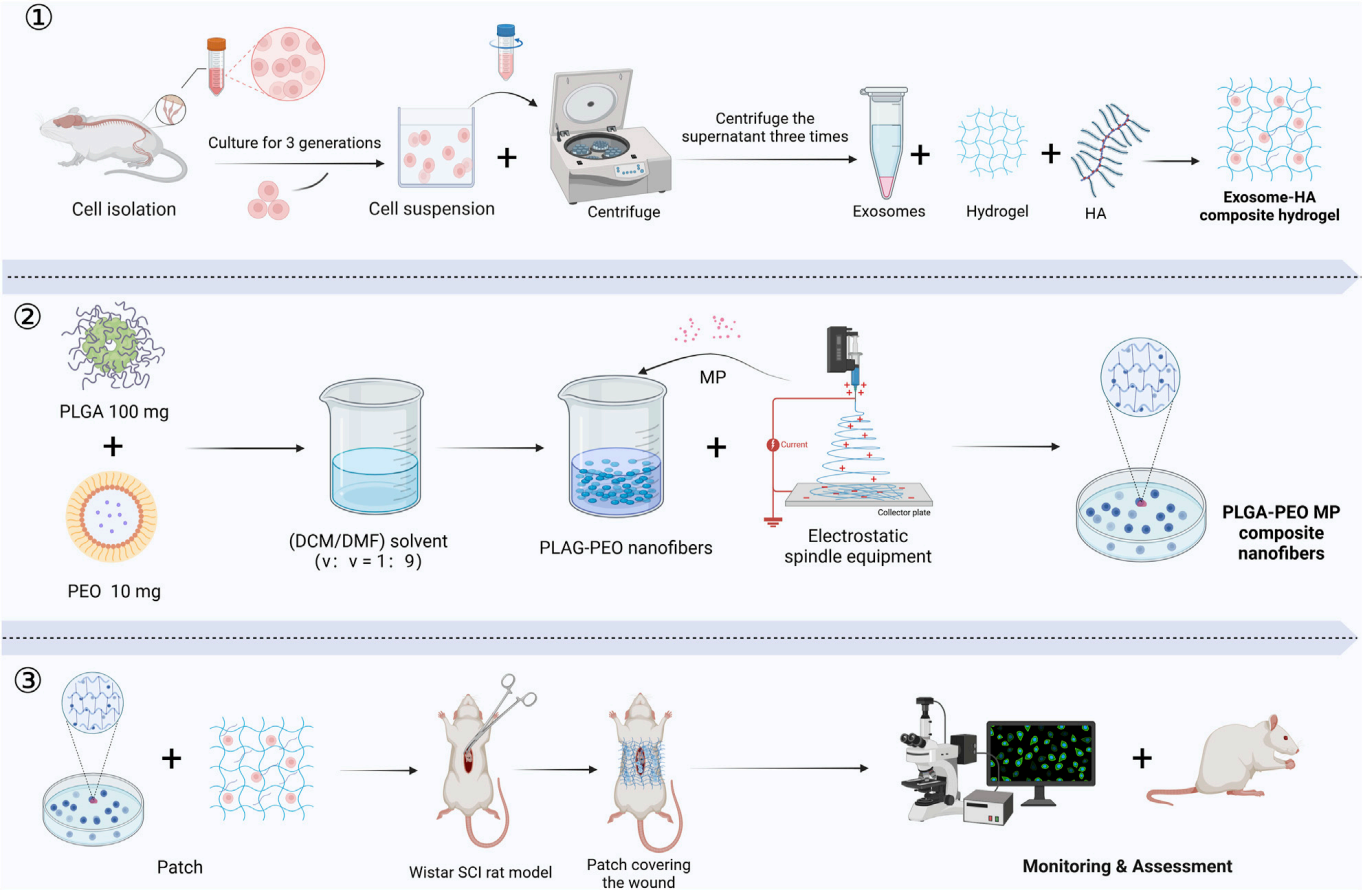
The production and application of an exosome-HA composite hydrogel patch and methylprednisolone-loaded nanofibers for promoting SCI repair: ① Exosome-Hyaluronic Acid Composite Hydrogel Preparation. ② Methylprednisolone-Loaded Nanofiber Production. ③ Application and Assessment in SCI Model. (MP, methylprednisolone; PLGA, poly (lactic-co-glycolic acid); PEO, poly (ethylene oxide); HA, hyaluronic acid; DCM, dichloromethane; DMF, N,N-dimethylformamide) (Created in https://BioRender.com).

Owing to their high plasticity, iPSCs offer new opportunities for SCI repair. Chen et al. demonstrated that tuning the viscoelasticity of hyaluronic acid hydrogels influenced patterning and vascularization of iPSC-derived spinal organoids. Softer hydrogels better mimicked the neural developmental microenvironment, promoting NSC-like cell differentiation (Chen et al., 202b). Wang et al. developed an injectable decellularized matrix hydrogel combined with exosomes derived from cortical neurons. This system inhibited neuronal apoptosis at the injury site and enhanced synaptic plasticity via miR-21-mediated regulation of the PTEN/AKT signaling pathway, ultimately promoting SCI repair (Wang et al., 202c). SCs primarily contribute to remyelination and axonal regeneration and are particularly valuable in SCI cases involving demyelination. Zhu et al. fabricated an SC-derived exosome-methylprednisolone composite patch. This system regulated the PI3K/Akt signaling pathway, reducing expression of the pro-apoptotic factor Caspase-3, thereby inhibiting neuronal apoptosis and promoting axonal regeneration (Zhu et al., 2023) (Figure 6). In summary, hydrogels not only provide essential support and protection for various stem cell types in SCI repair but also modulate inflammation, enhance neuro regenerative signaling, and improve the local microenvironment. These advances optimize cell-based therapies for SCI and offer a solid foundation for future precision stem cell transplantation strategies (Xu et al., 2024; Nie et al., 2024).

### 4.2 Hydrogel-based delivery systems for controlled release of drugs and bioactive molecules

Therapeutic agents, including nerve growth factors (NGFs), fibroblast growth factors (FGF), and anti-inflammatory drugs, effectively mitigate the inhibitory microenvironment post-SCI. However, conventional delivery methods face challenges such as short release durations, instability, rapid metabolism, and inadequate drug concentrations at the injury site (Meissner et al., 2024b), limiting their therapeutic potential. Hydrogels, with their flexibility, tunable degradation rates, excellent biocompatibility, and sustained-release capabilities, have emerged as optimal carriers for delivering drugs and bioactive agents in SCI therapy (Li et al., 202d).

Hydrogels enable controlled and sustained release of therapeutic agents through strategic structural design. For example, Thomas et al. developed hydrogels loaded with hepatocyte growth factor (HGF) that effectively inhibited scar formation, supported axonal growth, safeguarded respiratory neural circuits, and preserved neurological function following SCI (Thomas et al., 2024). Similarly, heparinized hydrogels encapsulating NT-3 optimized degradation kinetics and network architecture to ensure sustained release, preserve bioactivity, and extend *in vivo* efficacy, enabling continuous neural repair at the injury site (Meissner et al., 2024a).

Hydrogels also facilitate the co-delivery of multiple therapeutic agents, leveraging synergistic mechanisms to enhance the injury site microenvironment. For instance, thermosensitive hydrogels carrying minocycline effectively reduced local inflammation while promoting axonal regeneration (Gu et al., 2024). Bovine serum albumin (BSA)-based tissue-adaptive hydrogels enabled the dual release of PTX and bFGF, simultaneously inhibiting glial scar formation and promoting axonal growth (Zhu et al., 2024). Azithromycin, an anti-inflammatory agent, modulates macrophage polarization by promoting M2 macrophage gene expression while suppressing M1 macrophage gene expression. Wan et al. introduced an injectable hydrogel incorporating BM-MSCs and azithromycin, which effectively reduced inflammation and significantly enhanced neural network reconstruction (Wan et al., 2024).

### 4.3 Advancing mechanical properties and enhancing biocompatibility

Materials for SCI repair must possess excellent mechanical properties and biocompatibility to withstand the dynamic biomechanical environment and address the complex demands of tissue repair. Conventional hydrogel materials often fall short due to insufficient mechanical strength and unpredictable degradation rates (Chen et al., 2023). Recent advancements in enhancing the mechanical properties of hydrogels, such as tuning viscoelasticity and incorporating self-healing abilities, have significantly improved their functionality and adaptability. These innovations provide stable mechanical support, precise drug delivery, and superior tissue integration, thereby enhancing neural regeneration and advancing SCI repair strategies (Zhang et al., 2023).

Xiao et al. introduced a bioinspired injectable self-healing hydrogel with a dual-network architecture and dynamic crosslinking, which greatly enhanced mechanical stability, durability, and toughness. This hydrogel demonstrated excellent elasticity and recoverability under mechanical stress, reinforcing its strength and promoting neural regeneration. Similarly, Du et al. developed a composite hydrogel material incorporating silicon nitride and PVA, which exhibited exceptional damping capabilities and long-term mechanical stability, making it well-suited for SCI applications (Du et al., 2014). Chen et al. proposed a novel hydrogel material that hardens during swelling, improving mechanical stability while gradually adapting to the complex biomechanical requirements of the injury site. This innovative design significantly enhanced therapeutic outcomes in SCI compression models (Chen et al., 202c). Additionally, Correia et al. showcased a chitosan-based dynamically crosslinked hydrogel with optimized degradation and mechanical support properties, demonstrating significant efficacy in SCI models (Correia et al., 2024), Future efforts in hydrogel design should prioritize controllable degradation rates synchronized with tissue repair processes to minimize residual material-induced irritation and foster an optimal environment for tissue regeneration.

## 5 Challenges and constraints of hydrogels in spinal cord rehabilitation

Although hydrogels hold significant promise for spinal cord rehabilitation, their clinical translation and application face substantial challenges, particularly concerning material performance, functional design, microenvironment compatibility, and barriers to clinical adoption.

### 5.1 Limitations in material performance and functional stability

The material characteristics of hydrogels play a pivotal role in spinal cord rehabilitation; however, achieving a balance between mechanical robustness and functional stability poses significant challenges. For instance, hydrogels must possess sufficient mechanical strength to support tissue defects while maintaining flexibility and biocompatibility—a balance that remains difficult to achieve (Baek and Song, 2024). Moreover, conductive hydrogels face issues related to long-term stability and potential electrochemical side effects under electrical stimulation, underscoring the need for urgent solutions (Qazi et al., 2014). Over prolonged use, conductive hydrogels often experience reduced conductivity, compromising their durability (Zare et al., 2020). Advancing hydrogel material design to improve functional stability remains a critical area of investigation.

### 5.2 Deficiencies in micro-environmental compatibility and multi-functionality

SCI repair involves intricate microenvironmental modulation, encompassing inflammation, immune regulation, and neuronal regeneration. However, current hydrogel designs predominantly focus on single mechanisms, lacking the ability to achieve multifunctional synergy. For example, Yuan et al. observed that existing antioxidant hydrogels fail to effectively integrate anti-inflammatory and neuro-regenerative capabilities (Yuan et al., 2024; Sun et al., 2024). Furthermore, variations in SCI severity and immune responses among patients highlight the limitations of universal hydrogel designs, which do not address personalized therapeutic needs (Zhang et al., 202c). Future research should prioritize the development of multifunctional hydrogels capable of addressing the complexities of SCI microenvironments while accommodating patient-specific variability.

### 5.3 Challenges in bioactive factor delivery and clinical translation

Hydrogels often face challenges related to the instability of bioactive factors and the lack of precise control over their release during delivery. For instance, Thomas et al. reported that hydrogels struggle to regulate the release rate of neurotrophic factors such as BDNF, resulting in either excessive or insufficient local concentrations (Thomas et al., 2024). Moreover, active factors like exosomes may undergo partial inactivation during hydrogel preparation, significantly diminishing their reparative efficacy (Lu et al., 202b). In addition to these technical challenges, the clinical adoption of hydrogels is hindered by translational gaps between animal model findings and human outcomes, complex manufacturing processes, high production costs, and stringent regulatory approval requirements (Qiu et al., 2023). These obstacles highlight the urgent need for material optimization and technological advancements to facilitate the large-scale clinical implementation of hydrogels. By addressing these three critical challenges—bioactive factor stability and release, manufacturing scalability, and regulatory hurdles—future research can drive both technological innovations and clinical applications of hydrogels, paving the way for their effective use in spinal cord rehabilitation.

## 6 Prospects and future directions for hydrogels in spinal cord repair

Considering the challenges faced by hydrogels in spinal cord rehabilitation, future innovations must address several critical aspects. First, a multifunctional and integrated design is essential. By combining diverse bioactive functionalities with intelligent responsive materials (e.g., temperature, pH, enzyme triggers), hydrogels can better meet the intricate demands of spinal cord repair (Du et al., 2023; Zhao et al., 2023). Second, functionalized designs for precise drug delivery are paramount. Advanced hydrogels should incorporate nanotechnology-driven controlled-release systems (Jafarimanesh et al., 2023; Li et al., 2021) and dynamic multi-drug delivery platforms, effectively addressing the pharmacological needs across various SCI stages (Kong et al., 2023; Zhu et al., 2023). Third, the personalization and tunability of hydrogels hold great promise. By leveraging 3D printing technologies integrated with patient-specific data, future hydrogel systems can be tailored to individual injury profiles (Urciuolo et al., 2023; Chen et al., 2023; Song et al., 2023). These bespoke designs can dynamically adjust degradation rates and mechanical properties to synchronize with the healing process. Fourth, interdisciplinary integration is critical for advancing hydrogel technologies. Combining gene therapy with physical therapy, for example, can enhance synergistic effects. Conductive hydrogels paired with wireless electrical stimulation can restore neural signal transmission and facilitate neural network reconstruction (Zhang et al., 2023). Additionally, integrating gene-editing technologies like CRISPR-Cas9 into hydrogels enables localized modulation of inflammatory factor expression, significantly improving SCI repair efficiency (Song et al., 2023; Williams et al., 2024).

Lastly, prioritizing the translation and clinical application of hydrogels is vital. Future research should emphasize large animal models to evaluate long-term therapeutic efficacy Bioactive hydrogel-based organ printing techniques have shown promise in guiding the development and functional assessment of spinal organoids, providing valuable insights for clinical implementation (Urciuolo et al., 2023). Moreover, advancements in scalable injectable hydrogel systems and modular particulate hydrogels (Ross et al., 2024) are expected to reduce production costs and improve clinical practicality (Wang et al., 202b). In summary, the future trajectory of hydrogel applications in SCI therapy will emphasize intelligent, multifunctional, personalized, and clinically translatable advancements. By integrating precise drug delivery systems, tailored designs, and interdisciplinary innovations, hydrogels are poised to become a pivotal platform for spinal cord rehabilitation. Multifunctional hydrogel systems that merge gene therapy and physical therapy hold particular promise for delivering efficient and comprehensive solutions for neural repair.

## 7 Conclusion

SCI, a complex neurological condition, remains challenging to treat effectively due to its intricate anatomical and pathophysiological features. However, hydrogels, with their exceptional attributes, have addressed several limitations of conventional treatments, demonstrating significant potential in SCI repair. By mimicking the architecture of the ECM, hydrogels provide mechanical support to injured sites while enabling precise delivery and sustained release of drugs, bioactive molecules, and cells. This approach substantially improves the local microenvironment, promoting neuronal regeneration, angiogenesis, and the repair of the blood-spinal cord barrier. Additionally, conductive hydrogels restore neural electrical signal conduction, enhancing synaptic connectivity and facilitating neural functional recovery.

While hydrogel research has made significant progress, several critical challenges remain, including swelling effects, biotoxicity, limited functional axonal regeneration, and obstacles in translating hydrogel applications to human clinical use. Future research should focus on refining hydrogel synthesis techniques, enhancing stability, and improving scalability for mass production. Efforts should also prioritize elucidating signaling pathway mechanisms in SCI repair, developing intelligent materials responsive to microenvironmental changes, and conducting rigorous preclinical evaluations of toxicity and long-term efficacy. But with their multifunctionality and adaptability, hydrogels offer great potential for SCI repair. As material science and bioengineering progress, the development of multifunctional, personalized, and intelligent hydrogels is expected to transform SCI treatment, providing precise and efficient therapeutic solutions while offering renewed hope for functional recovery.
